# Impact of the selective A2_A_R and A2_B_R dual antagonist AB928/etrumadenant on CAR T cell function

**DOI:** 10.1038/s41416-022-02013-z

**Published:** 2022-10-20

**Authors:** Matthias Seifert, Mohamed-Reda Benmebarek, Daria Briukhovetska, Florian Märkl, Janina Dörr, Bruno L. Cadilha, Jakob Jobst, Sophia Stock, David Andreu-Sanz, Theo Lorenzini, Ruth Grünmeier, Arman Oner, Hannah Obeck, Lina Majed, Dario Dhoqina, Manouk Feinendegen, Adrian Gottschlich, Jin Zhang, Ulrike Schindler, Stefan Endres, Sebastian Kobold

**Affiliations:** 1 Center of Integrated Protein Science Munich (CIPS-M) and Division of Clinical Pharmacology, Department of Medicine IV, University Hospital, Ludwig-Maximilians-Universität München, Munich, Germany; 2 National Cancer Institute (NCI), Bethesda, MD USA; 3 Department of Medicine III, University Hospital, Ludwig-Maximilians-Universität München, Munich, Germany; 4 German Cancer Consortium (DKTK), Partner Site Munich, Munich, Germany; 5 Independent Consultant, Freiburg, Germany; 6 Einheit für Klinische Pharmakologie (EKLiP), Helmholtz Zentrum München, Research Center for Environmental Health (HMGU), Neuherberg, Germany

**Keywords:** Cancer immunotherapy, T cells

## Abstract

**Background:**

Chimeric antigen receptor (CAR) T cell therapy has been successfully translated to clinical practice for the treatment of B cell malignancies. The suppressive microenvironment of many malignancies is a bottleneck preventing treatment success of CAR T cells in a broader range of tumours. Among others, the immunosuppressive metabolite adenosine is present in high concentrations within many tumours and dampens anti-tumour function of immune cells and consequently therapeutic response.

**Methods:**

Here, we present the impact of the selective adenosine A2_A_ and A2_B_ receptor antagonist AB928/etrumadenant on CAR T cell cytokine secretion, proliferation, and cytotoxicity. Using phosphorylation-specific flow cytometry, we evaluated the capability of AB928 to shield CAR T cells from adenosine-mediated signalling. The effect of orally administered AB928 on CAR T cells was assessed in a syngeneic mouse model of colon carcinoma.

**Results:**

We found that immunosuppressive signalling in CAR T cells in response to adenosine was fully blocked by the small molecule inhibitor. AB928 treatment enhanced CAR T cell cytokine secretion and proliferation, granted efficient cytolysis of tumour cells in vitro and augmented CAR T cell activation in vivo.

**Conclusions:**

Together our results suggest that combination therapy with AB928 represents a promising approach to improve adoptive cell therapy.

## Background

Immunotherapy has become a new pillar of cancer therapy improving the clinical outcome of many patients with solid and haematological malignancies. Immune checkpoint blockade (ICB) has changed clinical practice and demonstrated the clinical utility of T cells in oncology [1]. ICB can lead to durable clinical responses in a variety of cancer types by reactivating suppressed or exhausted T effector cells [2, 3]. Currently, approved checkpoint inhibitors target mainly the programmed cell death protein 1 (PD-1) axis or cytotoxic T-lymphocyte-associated protein 4 (CTLA-4) to reinvigorate anti-tumour immunity [1]. However, a significant number of patients will either fail to respond or relapse after an initial response. Frequently, this can be attributed to an immunosuppressive environment, which is not overcome by conventional ICB [4]. Extracellular adenosine acts as soluble immune checkpoint and has emerged as a promising target for immunotherapy [5–7]. Within solid tumours, extracellular ATP levels are elevated due to high cellular turnover and active secretion [8, 9]. In the canonical pathway of extracellular adenosine generation, the ectonucleotidases CD39 and CD73 lead to the sequential dephosphorylation of extracellular ATP [10]. Expression of CD39 and CD73 on tumour cells, immune cells, fibroblasts, endothelial cells, and stromal cells is upregulated by hypoxia and TGF-β in the tumour microenvironment (TME) [6, 11, 12]. Other mechanisms such as extracellular AMP generation from NAD^+^ via CD38 and dysregulation of adenosine consuming pathways further contribute to extracellular adenosine accumulation within solid tumours [7, 13]. High concentrations of extracellular adenosine dampen anti-tumour immunity [5, 6, 14]. Upon binding of extracellular adenosine, the G-protein coupled adenosine A2_A_ and A2_B_ receptors (A2_A_R and A2_B_R) (K_d_ of 100 nM and 15 µM, respectively) [15] mediate an intracellular build-up of cAMP that compromises T cell effector functions [14, 16–19]. Owing to the predominant expression and higher affinity of A2_A_R, T cell suppression is primarily mediated by signalling downstream of A2_A_R [20, 21]. Besides direct suppression of effector cells, A2_A_R and A2_B_R activation promotes the generation and suppressive capacity of myeloid cells and regulatory T cells (T_regs_) [22–26].

Several agents counteracting the immunosuppressive adenosine axis have been developed and have shown promising preclinical anti-tumour activity [5–7]. The small molecule AB928/etrumadenant (for short, AB928) is a highly selective antagonist, targeting the A2_A_ and A2_B_ receptor [27, 28]. Importantly, results from early clinical trials in healthy volunteers and in patients demonstrated safety and favourable pharmacological properties of the new drug, potentially hinting at its potential for combinatorial treatments [27, 29, 30].

Another immunotherapeutic axis leveraging T cell function at the forefront of development in oncology are chimeric antigen receptor (CAR) T cells. These are autologous T cells, genetically engineered to stably express a synthetic receptor targeting a specific antigen [31]. While CAR T cell therapy is highly efficacious in the treatment of some haematological malignancies [32–34], it still lacks efficacy in the vast majority of tumours [35, 36]. Three major mechanisms for CAR T cell failure in solid tumours have been identified: Lack of T cell access to tumour sites, antigen heterogeneity and importantly immune suppression [37]. In fact, the immunosuppressive TME limits CAR T cell responses against solid tumours [31] and anti-tumour CAR T cell responses are suppressed by adenosine. Recent preclinical evidence suggests that pharmacological as well as genetic targeting of A2_A_R may improve CAR T cell efficacy [20, 21, 38–40]. However, targeting has thus far been limited to the A2_A_R pathway and mainly to anti-CD19, anti-mesothelin and anti-Her2 CAR T cells [20, 21, 38–40]. Whether A2_B_R co-targeting yields similar or better results is unclear. Given the high complexity of CAR function and design [36], it also remains to be determined if the approach is broadly applicable across different models. These considerations and the advanced clinical development stage of AB928 become important when considering implementing adenosine receptor blockade into cell therapy trials. Thus, we asked the question if AB928-mediated blockade of A2_A_R and A2_B_R synergises both with murine and human CAR T cells for optimised functionality against a range of murine and human cancer cell lines.

## Materials and methods

### Mice

Wild-type C57BL/6 and BALB/c mice were purchased from Janvier (St. Bertevin, France) or Charles River (Sulzfeld, Germany).

### Animal experiments

All experimental studies were approved and performed in accordance with guidelines and regulations of the local regulatory agency (Regierung von Oberbayern). The experiments were randomised and conducted with adequate controls. The investigators were not blinded during the experiments. Tumours were induced by subcutaneous injection of 10^6^ CT26-EpCAM tumour cells. Daily oral treatment with 10 mg AB928 formulated in 100 µl PEG/solutol (70/40) or control treatment was initiated, once the tumour was palpable. Mice were injected intravenously with 10^7 ^T cells the following day. In accordance with the animal experiment application, tumour growth and health status of mice were checked at least every other day.

### Cell lines

Murine Panc02-EpCAM, 4T1, T110299 and CT26-EpCAM have been previously described [41–43]. Murine LL/2 were purchased from the European Collection of Authenticated Cell Cultures (ECACC). The T110299 and LL/2 cell lines were modified to stably express full-length murine EpCAM (UNIPROT entry Q99JW5), to generate the cell lines T110299-EpCAM and LL/2-EpCAM. Human SUIT-2-MSLN have been previously described [41]. 293Vec-Galv, 293Vec-Eco, and 293Vec-RD114 have been previously described [44]. The virus producing cell lines 293Vec-Eco for anti-EpCAM-CAR-28z and 293Vec-RD114 for anti-MSLN-CAR-28z have been previously described [45]. 293Vec-RD114 for anti-MSLN-CAR-4-1BBz were generated as previously described [46]. All cell lines were cultured as previously described [41, 42]. All cell lines were periodically tested for mycoplasma contamination with the commercial testing kit MycoAlert^TM^ (Lonza, Basel, Switzerland). Authentication of human cell lines by short tandem repeat DNA profiling was conducted in-house.

### Murine T cell culture and transduction

The transduction using the retroviral vector pMP71 and culture of primary murine T cells has been previously described [47]. In brief, 1.2 × 10^6^ virus producing 293Vec-Eco cells were seeded into a 6-well plate 24 h prior to splenocyte isolation. After 48 and 72 h, the virus-containing supernatant was used to transduce murine T cells. Murine T cells were expanded from murine splenocytes by activation with anti-CD3 and anti-CD28 antibodies (clones 145-2C11 and 37.51, Thermo Fisher Scientific, Waltham, MA, USA) and human IL-2 (10 IU/ml, Novartis, Basel, Switzerland) for 24 h. Subsequently murine T cells were stimulated Dynabeads™ Mouse T-Activator CD3/CD28 (Thermo Fisher Scientific) and human IL-15 (50 ng/ml, Peprotech). Prior to some experiments Dynabeads™ were removed.

### Human T cell culture and transduction

The transduction using the retroviral vector pMP71 and culture of primary human T cells has been previously described [48]. In brief, 1.2 × 10^6^ virus producing 293Vec-RD114 cells were seeded into a 6-well plate and virus containing supernatant was harvested and used for transduction after 48 and 72 h. After approval by the institutional review board of the Ludwig-Maximilians-Universität (Munich, Germany), peripheral blood samples were collected from healthy donors. Peripheral blood mononuclear cells (PBMC) were isolated by density gradient centrifugation using Histopaque®-1077 (Sigma-Aldrich, St. Louis, MO, USA). T cells were isolated from PBMCs by magnetic cell separation with CD3 Microbeads (Miltenyi Biotec, Bergisch Gladbach, Germany). T cells were activated with Dynabeads™ Human T-Activator CD3/CD28 (Thermo Fisher Scientific) and T cell culture was supplemented with human IL-2 (200 IU/ml) and human IL-15 (5 ng/ml). Prior to experiments Dynabeads™ were removed.

### Flow cytometry

Multicolour flow cytometry was carried out according to previously published protocols [49]. Samples were analysed with a CytoFLEX LX flow cytometer (Beckmann Coulter, Brea, CA, USA) and BD FACSCanto™ II and BD LSRFortessa™ II flow cytometers (BD Biosciences, Franklin Lakes, NJ, USA). Cells were stained with Fixable Viability Dye eFluor™ 780 (Thermo Fisher Scientific) to exclude dead cells. Surface staining of murine T cells was performed with the following antibodies: anti-CD3 (BV510 or BV421, clone 17A2, Biolegend San Diego, CA, USA), anti-CD4 (BV785 or AF700, clone GK1.5, Biolegend), anti-CD8a (Pacific Blue or FITC, clone 53-6.7, Biolegend), anti-CD25 (APC, clone PC61, Biolegend), anti-CD44 (PerCP/Cy5.5, clone IM7, Biolegend), anti-CD69 (PE-Cy7 or BV510, clone H1.2F3, Biolegend), anti-CD62L (Pacific Blue or PE/Cy5, clone MEL-14, Biolegend), anti-PD-1 (BV421 or BV650, clone 29F.1A12, Biolegend), anti-TIM3 (BV605 or APC, clone RMT3-23, Biolegend), anti-LAG3 (PerCP/Cyanine5.5, clone C9B7W, Biolegend), anti-TIGIT (APC, clone 1G9, Biolegend). Anti-EpCAM CAR expression on T cells was confirmed by mCherry tag detection. Surface staining of human T cells was performed with the following antibodies: anti-CD3 (PerCP, clone OKT3, Biolegend), anti-CD4 (AF700, clone A161A1, Biolegend), anti-CD8 (BV785, clone RPA-T8, Biolegend), anti-CD45RO (PE-Cy7, clone UCHL1, Biolegend), anti-CCR7 (BV412, clone G043H7, Biolegend), anti-PD-1 (APC, clone EH12.2H7, Biolegend), anti-TIM3 (PE/Dazzle™ 594, clone F38-2E2, Biolegend), anti-LAG3 (BV605, 11C3C65, Biolegend). Anti-MSLN-28z CAR or Anti-MSLN-4-1BBz CAR expression on T cells was detected by staining for the c-myc tag included in the receptors using anti-c-myc (FITC, clone SH1-26E7.1.3, Miltenyi Biotec). For intracellular staining, anti-EpCAM CAR T cells were stimulated by plate bound recombinant EpCAM-Fc chimera protein (1 µg/ml coated overnight, R&D Systems, Minneapolis, MN, USA) for 18 h. For the last 4 h BD GolgiStop™ (BD Biosciences) was added. Cells were fixed and permeabilised using BD Cytofix/Cytoperm™ (BD Biosciences) and subsequently stained for IFN-γ (PE-Cy7, clone XMG1.2, Biolegend). For phospho-specific flow cytometry of p-CREB, T cells were pretreated with AB928 (titration from 10 nM to 10 µM) for 1 h, then 5 µM NECA was added for 1 h. Cells were fixed and permeabilised using BD Cytofix™ and BD Phosflow™ Perm Buffer III (both BD Biosciences) according to the manufacturer’s instructions. Cells were then stained for CREB (pS133) / ATF-1 (pS63) (AF647, Clone J151-21, BD Biosciences).

### Cytotoxicity assay

Impedance-based real-time killing assays were performed using an xCELLigence system (ACEA Biosciences, San Diego, CA, USA), as previously described [41]. Briefly, 2.5 ×10^4^ Panc02-EpCAM, 4T1, or SUIT-2-MSLN tumour cells were seeded per well in a 96-well plate. A total of 5 ×10^4^ anti-EpCAM CAR T cells or 2.5 ×10^4^ anti-MSLN-28z CAR T cells and the indicated treatments were added to the tumour cells when the cell index reached approximately 1. The cell index is a measure of the relative change in the electrical impedance to represent the cell status and was normalised to the timepoint of treatment.

### Cytokine and granzyme B release assay

CAR T cells were treated and stimulated as indicated in the figure legends. Protein concentrations in the supernatant were determined by commercially available ELISA (human and murine IFN-γ and IL-2 by BD Biosciences and murine TNF-α and murine granzyme B (GzmB) by R&D Systems).

### Proliferation assay

In a 96-well plate, 10^5^ anti-EpCAM CAR T cells per well were activated by plate bound recombinant EpCAM-Fc chimera protein (0.5 µg/ml coated overnight, R&D Systems) over a period of 48 h. Before the experiment Dynabeads™ were removed. Cell numbers were determined by flow cytometry with CountBright™ Absolute Counting Beads (Thermo Fisher Scientific) at the beginning and end of the assay to calculate fold proliferation of T cells.

### Statistical analysis

The flow cytometry data were analysed with FlowJo V10.3 software. Statistical analysis was performed with the GraphPad Prism 9 software. Data are presented as indicated in the figure legends. Statistical analysis was performed as indicated in the figure legends. The Bonferroni correction was used to account for multiple comparisons. *P* < 0.05 was considered statistically significant and represented as *P < 0.05, **P < 0.01, and ***P < 0.001. No statistical methods were used to predetermine sample size.

## Results

### Adenosine inhibits CAR T cell activation

**Fig. 1:**
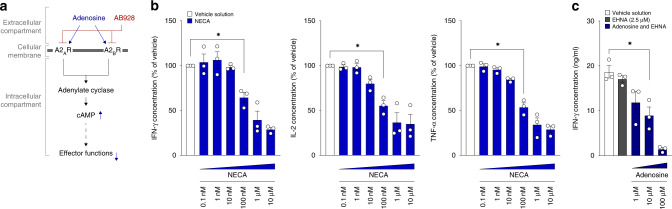
Adenosine inhibits CAR T cell activation. **a** Schematic illustration of the rationale of combining CAR T cell therapy and AB928. **b**, **c** In all, 2.5 × 10^4^ murine anti-EpCAM CAR T cells were cocultured with 2.5 × 10^4^ Panc02-EpCAM tumour cells for 24 h in the presence or absence of **b** NECA (titration from 0.1 nM to 10 µM) or **c** adenosine (titration from 1 µM to 100 µM + EHNA 2.5 µM). **b** IFN-γ, IL-2 and TNF-α concentrations in the coculture supernatant were determined by ELISA. Data were normalised to the vehicle control condition. **c** IFN-γ concentrations in the coculture supernatant were determined by ELISA. **b**, **c** Data are shown as mean ± SEM of *n* = 3 independent experiments with each dot representing the mean value of an individual experiment. *P < 0.05 by two-sided *t* test.

Extracellular adenosine suppresses T cell and CAR T cell activation [5, 7, 38]. We hypothesised that combination therapy with AB928 may enhance CAR T cell function by blocking immunosuppressive signalling in response to extracellular adenosine, thereby maintaining effective CAR T cell responses (Fig. 1a). To confirm the suppressive effect of adenosine in our murine anti-EpCAM CAR T cell model, we cocultured said CAR T cells with tumour cells of the pancreatic ductal adenocarcinoma cell line Panc02-EpCAM. Cocultures were performed in the presence or absence of the stable adenosine receptor agonist 5’-*N*-ethylcarboxamide adenosine (NECA) or adenosine (combined with erythron-9-(2-hydroxy-3-nonyl)adenine (EHNA) to prevent adenosine deaminase mediated degradation of adenosine) in serial titrations to mimic high concentrations of extracellular adenosine in the TME. After 24 h the coculture supernatants, representing T cell activation and degranulation, were collected, and subjected to ELISA readouts. As hypothesised, NECA dampened IFN-γ, IL-2 and TNF-α release in a dose dependent manner (Fig. 1b). Also, adenosine itself resulted in a decreased protein concentration of IFN-γ in the supernatants (Fig. 1c). These results are in concordance with previously published data and highlight the susceptibility of CAR T cells to adenosine-mediated suppression, supporting the rationale for studying the combination therapy with AB928 [20, 21, 38–40].

### AB928 protects CAR T cell activation from adenosine-mediated suppression

**Fig. 2:**
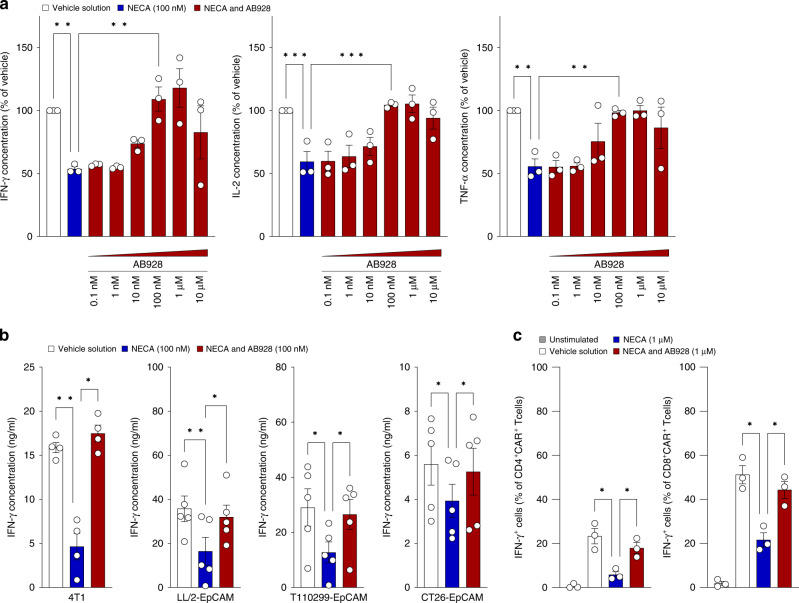
AB928 protects CAR T cells from adenosine mediated suppression. **a** In all, 2.5 × 10^4^ murine anti-EpCAM CAR T cells were cocultured with 2.5 ×10^4^ Panc02-EpCAM tumour cells for 24 h in the presence or absence of NECA (100 nM) and AB928 (titration from 0.1 nM to 10 µM). IFN-γ, IL-2 and TNF-α concentrations in the coculture supernatant were determined by ELISA. Data were normalised to the vehicle control condition. **b** Also, 2.5 ×10^4^ murine anti-EpCAM CAR T cells were cocultured with 2.5 × 10^4^ 4T1, LL/2-EpCAM, T110299-EpCAM and CT26-EpCAM tumour cells for 24 h cells in the presence or absence of NECA (100 nM) and AB928 (100 nM). Cytokine concentrations in the coculture supernatant were determined by IFN-γ ELISA. **c** Intracellular staining of murine anti-EpCAM CAR T cells activated with plate bound recombinant EpCAM for 18 h in the presence or absence of NECA (1 µM) and AB928 (1 µM). **a**–**c** Data are shown as mean ± SEM of **a**, **c** *n* = 3 or **b** *n* = 4–5 independent experiments with each dot representing the mean value of an individual experiment. *P < 0.05, **P < 0.01, ***P < 0.001 by one-way ANOVA.

To investigate whether AB928 can shield CAR T cells from adenosine-mediated suppression, we cocultured anti-EpCAM CAR T cells and Panc02-EpCAM tumour cells in the presence or absence of inhibiting concentrations of NECA and serially titrated AB928. While NECA impaired IFN-γ, IL-2 and TNF-α secretion as described above, addition of AB928 in concentrations ranging from 100 nM to 10 μM fully restored cytokine secretion (Fig. 2a). To rule out cell line-specific effects, cocultures were performed with a panel of murine cancer cell lines (namely the mammary carcinoma cell line 4T1, the lung carcinoma cell line LL/2-EpCAM, the pancreatic ductal adenocarcinoma cell line T110299-EpCAM and the colon carcinoma cell line CT26-EpCAM). The data (Fig. 2b) are consistent among all cell lines, corroborating the overarching principle of adenosine suppression and CAR T cell disinhibition by AB928.

Next, we used flow cytometry to analyse the effect of NECA and AB928 on anti-EpCAM CAR T activation upon stimulation with recombinant EpCAM. We found that NECA reduced the amount of IFN-γ^+^ cells in the population of CD4^+^ and CD8^+^ CAR T cells, whereas AB928 reversed NECA-mediated suppression (Figs. 2c and S1a). Activation markers CD25 and CD69 were downregulated after NECA treatment and AB928 reversed this effect, both for cocultures with antigen expressing tumour cells or recombinant EpCAM stimulation. Interestingly, upregulation of CD25 and CD69 in the AB928 containing condition was more pronounced than in the vehicle control condition when CAR T cells were activated by antigen-expressing tumour cells, but not when activated with recombinant protein (Fig. S1b). Overall, these findings demonstrate the capability of AB928 to counteract adenosine-mediated suppression of CAR T cells.

### AB928 enables efficient CAR T cell effector responses

**Fig. 3:**
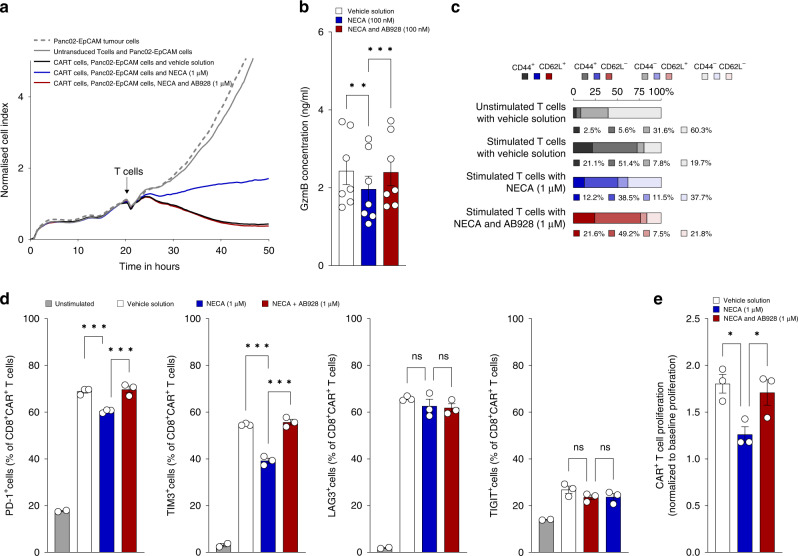
AB928 enables efficient CAR T cell effector responses in vitro. **a** RTCA of coculture with 5 × 10^4^ murine anti-EpCAM CAR T cells and 2.5 × 10^4^ Panc02-EpCAM tumour cells in the presence or absence of NECA (1 µM) and AB928 (1 µM). CAR T cells and treatments were added at the timepoint indicated by the arrow. **b** In all, 2.5 × 10^4^ murine anti-EpCAM CAR T cells were cocultured with 2.5 × 10^4^ Panc02-EpCAM tumour cells for 24 h in the presence or absence of NECA (100 nM) and AB928 (100 nM). GzmB concentrations in the coculture supernatant were determined by ELISA. **c**, **e** In all, 10^5^ murine anti-EpCAM CAR T cells were activated with plate-bound recombinant EpCAM for **c** 18 h or **e** 48 h in the presence or absence of NECA (1 µM) and AB928 (1 µM). **c** Phenotypic characterisation of CAR T cells by flow cytometry. **d** Also, 10^5^ murine anti-EpCAM CAR T cells were cocultured with 2.5 × 10^5^ CT26-EpCAM tumour cells. Expression of inhibitory receptors on CD8^+^CAR^+^ T cells by flow cytometry. **e** CAR T cell proliferation was determined by flow cytometry with counting beads at the beginning and end of the experiment. Data were normalised to baseline proliferation of unstimulated cells. **a**, **c** Representative experiment of *n* = 3 independent experiments. Data represents mean of technical replicates. **b**, **e** Data are shown as mean ± SEM of **b** *n* = 7 or **e** *n* = 3 independent experiments with each dot representing the mean value of an individual experiment. **d** Representative experiment of *n* = 3 independent experiments. Data are shown as mean ± SEM of technical replicates. *P < 0.05, **P < 0.01, ***P < 0.001 by one-way ANOVA.

To test if AB928 can improve the anti-tumour efficacy of CAR T cells, we performed cytotoxicity assays using real time cell analysis (RTCA). In the control condition, anti-EpCAM CAR T cells efficiently lysed Panc02-EpCAM tumour cells. Addition of inhibiting concentrations of NECA resulted in diminished tumour cell lysis, whereas AB928 rescued CAR T cell-mediated cytotoxicity (Fig. 3a). In line with this, AB928 also enabled the efficient cytolysis of 4T1 breast cancer cells, despite inhibiting concentrations of adenosine being present (Fig. S2a). Granzymes are important mediators of CAR T cell killing [50]. Consistent with our data on cytotoxicity, we observed that AB928 augmented GzmB release from CAR T cells in the presence of NECA (Fig. 3b). To determine the effect of NECA and AB928 on CAR T cell phenotype and proliferation, we performed flow cytometry. Staining for CD44 and CD62L expression, we observed a transition towards an effector-like (CD44^+^/CD62L^−^) CAR T cell phenotype upon stimulation. NECA-mediated drifting of this phenotype was blocked by AB928 (Figs. 3c and S2b). Activation-induced upregulation of the inhibitory receptors PD-1 and TIM3 was inhibited by NECA and restored by AB928. LAG3 and TIGIT expression was not influenced by NECA or AB928 (Fig. 3d). CAR T cell proliferation in the presence of inhibiting NECA concentrations was also augmented by AB928 (Fig. 3e). Thus, AB928 efficiently blocks adenosine-mediated suppression of crucial CAR T cell effector functions.

### AB928 shields CAR T cells from immunosuppressive signalling in response to adenosine

**Fig. 4:**
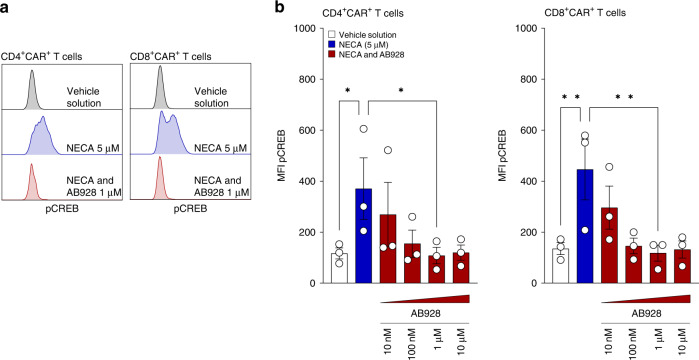
AB928 shields CAR T cells from immunosuppressive signalling in response to adenosine. **a**, **b** Phospho-specific flow cytometry of CREB of murine anti-EpCAM CAR T cells after treatment with NECA (5 µM) and/or AB928 (titration from 10 nM to 10 µM). Data are shown as **a** representative histogram and **b** mean ± SEM of *n* = 3 independent experiments with each dot representing the mean value of an individual experiment. *P < 0.05, **P < 0.01 by one-way ANOVA.

A2_A_R and A2_B_R signalling promotes the activity of adenylyl cyclases, leading to elevated levels of cAMP. Intracellular cAMP accumulation then activates protein kinase A, resulting in the phosphorylation of cAMP response element-binding protein (CREB) which promotes FoxP3 expression and thus T_reg_ generation [51, 52]. To investigate the effect of AB928 on adenosine-mediated signalling in anti-EpCAM CAR T cells, we performed phosphorylation-specific flow cytometry, staining for p-CREB. Likewise, basal p-CREB levels were increased in CD4^+^ and CD8^+^ CAR T cells upon incubation with saturating concentrations of NECA (5 μM). When preincubated with AB928 at concentrations higher than 1 μM, the addition of NECA did not result in any detectable increase in CREB phosphorylation (Fig. 4a, b). These data indicate that AB928 is capable of effectively shielding CAR T cells from immunosuppressive signalling even in the presence of high concentrations of extracellular adenosine.

### Orally administered AB928 augments CAR T cell activation in vivo

**Fig. 5:**
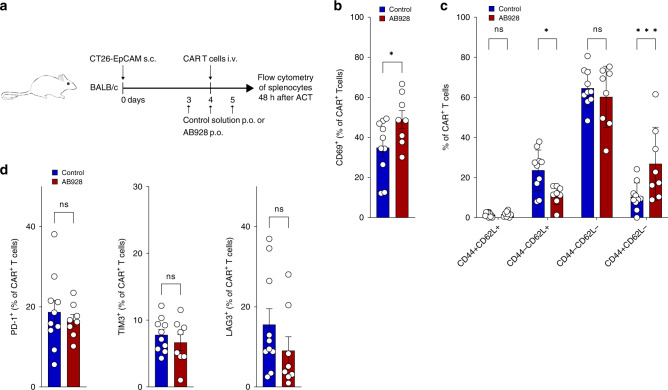
Orally administered AB928 augments CAR T cell activation in vivo. **a** Schematic illustration of the experimental setup. **b**–**d** CAR^+^ T cells in the spleen were tracked and phenotyped by flow cytometry at the end of the experiment. Data are shown as mean ± SEM of control *n* = 10 mice and AB928 *n* = 8 mice. *P < 0.05, ***P < 0.001 by two-sided *t* test.

We next evaluated the effect of AB928 on CAR T cells in vivo. BALB/c mice were subcutaneously injected with 10^6^ CT26-EpCAM tumour cells. Once tumours were established, daily oral treatment with 10 mg AB928 or control treatment was initiated. In all, 10^7^ anti-EpCAM CAR T cells were intravenously injected the following day. Forty-eight hours later, the CAR T cell phenotype was assessed by flow cytometry (Fig. 5a). In line with our previous findings, AB928 treatment resulted in increased expression of CD69, and CAR T cells presented a more effector-like (CD44^+^/CD62L^−^) phenotype (Fig. 5b, c). Expression of the inhibitory receptors PD-1, TIM3, and LAG3 was not influenced by AB928 treatment (Fig. 5d). These results indicate that orally administered AB928 boosts CAR T cell activation in vivo.

### AB928 ameliorates activation of human CAR T cells in the presence of adenosine

**Fig. 6:**
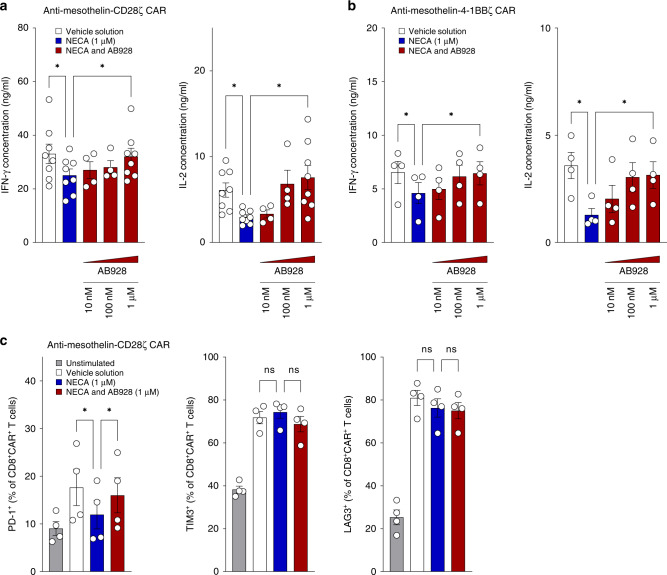
AB928 ameliorates activation of human CAR T cells in the presence of adenosine. Coculture of 2.5 × 10^4^ human **a** anti-MSLN-CD28z CAR T cells or **b** anti-MSLN-4-1BBz CAR T cells and 2.5 × 10^4^ SUIT-2-MSLN tumour cells in the presence or absence of NECA (1 µM) and AB928 (titration from 10 nM to 1 µM). **a**, **b** After 24 h, IFN-γ and IL-2 concentrations in the coculture supernatant were determined by ELISA. **c** Coculture of 10^5^ anti-MSLN-CD28z CAR T cells and 2.5 × 10^4^ SUIT-2-MSLN tumour cells in the presence or absence of NECA (1 µM) and AB928 (1 µM). After 48 h, phenotype of CD8^+^ CAR^+^ T cells was determined by flow cytometry. **a**–**c** Data are shown as mean ± SEM of *n* = 4–8 independent experiments with each dot representing the mean value of an individual experiment. *P < 0.05, **P < 0.01 by one-way ANOVA.

We generated human CAR T cells expressing a second-generation anti-mesothelin (MSLN) CAR with an intracellular CD3ζ domain and either a CD28 (anti-MSLN-28z CAR) or 4-1BB (anti-MSLN-4-1BBz CAR) costimulatory domain (Fig. S3) to confirm AB928 effects in different CAR designs in the human system. Anti-MSLN CAR T cells were cocultured with SUIT-2-MSLN tumour cells in the presence or absence of NECA and serially titrated AB928. NECA dampened IFN-γ and IL-2 release of both anti-MSLN-28z (Fig. 6a) and anti-MSLN-4-1BBz (Fig. 6b) CAR T cells. Interestingly, IFN-γ release was affected by NECA to a lesser extent than IL-2. AB928 in turn restored cytokine release (Fig. 6a, b). Thus, AB928 blocks adenosine mediated suppression of cytokine production by human CAR T cells, independently of CAR design. To further analyse the effect of NECA and AB928 on human anti-MSLN-28z CAR T cells, we determined the CAR T cell phenotype by flow cytometry and performed RTCA-based cytotoxicity assays. AB928 restored activation dependent upregulation of PD-1, whereas TIM3, LAG3 and CD45RO/CCR7 expression were not influenced by NECA or AB928 (Figs. 6c and S4b). Neither NECA nor AB928 modulated CAR T cell cytotoxicity of anti-MSLN-28z CAR T cells (Fig. S4a).

## Discussion

Among other aspects, insufficient T cell trafficking to the tumour [53, 54], antigen heterogeneity [46] and the immunosuppressive TME [49] are major factors preventing treatment success of CAR T cell therapy in a broader range of tumours [35, 37]. Extracellular adenosine acts as a soluble immune suppressant in the TME, limiting effective anti-tumour T cell responses [14]. Many strategies targeting the adenosine axis have been developed and investigated. Among others, targeting the adenosine-producing ectonucleotidases CD39 and CD73 and the adenosine receptors A2_A_R and A2_B_R to improve the efficacy of immunotherapies or conventional chemotherapies has been explored [5, 6]. Blockade of A2_A_R has been brought forward as a strategy to enhance CAR T cells but it remained unclear if co-blockade of A2_B_R would yield similar or better results and if the effect would be conserved across different CAR designs. In the present study, the impact of the A2_A_R and A2_B_R antagonist AB928 on efficacy of different second-generation CAR T cells was assessed.

It has been described that extracellular adenosine specifically acts by dampening T cell receptor-mediated signalling [55]. This lack of adequate T cell stimulation is partly responsible for diminished effector functions, T cell anergy and generation of T_regs_ in the presence of high concentrations of adenosine [22]. In our model of murine anti-EpCAM CAR T cells, cytokine secretion, upregulation of activation markers and proliferation in response to antigen-dependent stimulation were markedly impaired in the presence of adenosine or its analogue NECA. In contrast, addition of AB928 led to improved IFN-γ, IL-2 and TNF-α secretion as well as restored upregulation of PD-1, TIM3, CD25 and CD69 in response to antigen-stimulus, indicating adequate T cell activation. Beyond being a surrogate marker for T cell functionality, this is of critical importance as IFN-γ and TNF-α have been described to play a critical role for treatment success of ACT against solid tumours by inducing anti-tumour immunity from bystander immune cells and by evoking antigen-independent destruction of tumour and stroma cells [56–59]. Similarly, IL-2 promotes T cell proliferation and effector functions [60, 61]. Consistently, AB928 also reversed NECA mediated inhibition of T cell proliferation. We observed that IFN-γ production of both CD4^+^ and CD8^+^ CAR T cells was enhanced in the presence of AB928. This finding is of interest considering recent findings highlighting the significant role played by CD4^+^ T cell in establishing and sustaining anti-tumour immunity [61–64]. While the effect on PD-1 and TIM3 expression is in line with results by Giuffrida et al. [21], it contrasts some previously reported data suggesting that adenosine signalling enhances exhaustion and anergy of T cells, evidenced by the upregulation of checkpoint molecules [20, 65]. However, in our experimental setting, there was no chronic stimulation, thus anergy and exhaustion were unlikely to occur. Instead, upregulation of PD-1 and TIM3 is a physiological consequence of acute T cell activation [66, 67] and in consequence of adenosine-mediated inhibition of T cell activation any markers associated with activation will be reduced and likewise reinstalled upon inhibition of adenosine signalling.

Previous studies have extensively characterised the effect of adenosine on adoptively transferred T cells [14, 21, 38]. Importantly, genetic ablation of adenosine receptors leads to an effector-like phenotype, enhanced activation, and effector function of CAR T cells, ultimately resulting in better survival [21, 38]. Here we found that daily oral dosing of AB928 is efficacious in improving CAR T cell activation and promotes an effector-like phenotype of CAR T cells in tumour bearing mice, confirming previous findings, and demonstrating functionality of the adenosine receptor inhibitor on CAR T cells in an in vivo setting.

Moreover, AB928 also protected second-generation human anti-MSLN-28z and anti-MSLN-4-1BBz CAR T cells from adenosine-mediated suppression of cytokine release. Of note, cells bearing second-generation CARs with different costimulatory motives for the same antigen were comparably suppressed by adenosine, indicating that neither CD28 nor 4-1BB costimulatory domains can overcome adenosine-mediated effects.

The effect of adenosine on direct CAR T cell killing is controversial. Masoumi et al. observed reduced cytolytic function of human CAR T cells in the presence of NECA in a flow cytometry-based cytotoxicity assay [39]. Interestingly A2_A_R knockdown, but not SCH58261, a small molecule A2_A_R antagonist, protected CAR T cells. Here we used RTCA to assess CAR T cell killing. We observed impaired cytotoxic function of murine anti-EpCAM CAR T cells in the presence of NECA or adenosine. Of note, AB928 protected CAR T cell killing capacity from inhibition, highlighting its potential utility. This also calls attention to the advantages that dual targeting of adenosine receptors might have over single targeting, although further work is still required to fully demonstrate this observation. In contrast, we did not observe suppression of human anti-MSLN-28z CAR T cell cytotoxicity by NECA. Importantly, this is in line with previously published evidence by Beavis et al. and Giuffrida et al. reporting that NECA had negligible impact on murine and human CAR T cell cytotoxicity in a chromium release assays [21, 38]. Thus, it seems, that adenosine does not hamper the killing abilities of CAR T cells in certain settings, possibly because of the strength of CAR T cells in vitro. However, we cannot disregard the possibility that in other settings, such as long-term exposure to adenosine, the cytotoxic potential of these CAR T cells would be suppressed by adenosine. Overall, the factors determining to what extend adenosine influences CAR T cell cytotoxicity have yet to be defined.

T cells are highly sensitive to adenosine-mediated suppression, with Giuffrida et al. recently suggesting that genetic or pharmacological targeting of A2_A_R should prevent more than 50% of the adenosine-mediated effect on T cells [21]. A2_A_R and A2_B_R activation leads to signalling via the cAMP-PKA-CREB axis [51]. We have shown that NECA-induced CREB phosphorylation in anti-EpCAM CAR T cells was abrogated in the presence of 1 µM AB928. This result demonstrates that AB928 can effectively and efficiently block immunosuppressive signalling in response to adenosine. Importantly, AB928 plasma levels of 1 µM and higher are feasible and safe in patients [27]. We found that signalling was blocked in both CD4^+^ and CD8^+^ CAR T cells. This is important, as it has been previously shown that A2_A_R and A2_B_R activation on CD4^+^ T cells may promote the generation of T_regs_ [23, 26]. Putatively CAR-transduced T_regs_ [68] could be potentially boosted by adenosine but not so if AB928 is present, although this would need to be formally demonstrated.

It remains to be determined how AB928 compares to other strategies targeting adenosine receptors to overcome suppression of adoptively transferred T cells. The immunosuppressive effect of adenosine on T cells is primarily mediated by the predominantly expressed A2_A_R, making it an attractive target to improve T cell-mediated anti-tumour immunity [20, 21]. The small molecule A2_A_R antagonists SCH58261 [38–40], CPI-444 [65] and KW6002 [69], as well as approaches genetically targeting A2_A_R with shRNA knockdown [38, 39] or CRISPR/Cas9-mediated knockout [20, 21] have successfully been used to enhance ACT in preclinical studies. However, growing evidence suggests that A2_B_R also plays an important role in adenosine-mediated suppression of anti-tumour responses by indirectly suppressing T cell function. It has been shown, that A2_B_R antagonism reduces differentiation and suppressive capacity of T_regs_ and suppressive myeloid cells, leading to an increased presence of tumour-infiltrating CD8^+^ T cells in vivo [23, 25, 26, 70]. Recently, Chen et al. showed that pharmacological A2_B_R antagonism prior to adoptive T cell transfer improves treatment efficacy [71]. Among small molecule inhibitors targeting adenosine receptors, AB928 is the first dual A2_A_R and A2_B_R antagonist in clinical development. However, we have not yet formally proven the advantage of dual over single targeting.

Genetic targeting is an elegant way to render CAR T cells resistant to one or more immunosuppressive factors [72]. It provides continuous protection from suppression and therefore allows single dosing of the T cell product. However, safety concerns regarding off-target editing and the administration of CAR T cells with permanent deletion of immune checkpoints remain [72]. Small molecule inhibitors in turn may suffer from variable pharmacokinetics (PK) and require repeated dosing, which in turn can be beneficial in case of unwanted serious adverse events [73]. Further, they may also enable improved recruitment of endogenous anti-tumour responses by acting on other immune cells [74, 75]. The combination therapy of CAR T cells with cell intrinsic or extrinsic ICB has already been explored in more detail for the PD-1 axis and is currently being investigated in clinical trials. Thus far it is unclear which approach provides the best results regarding clinical outcome and safety [76].

AB928 is currently under evaluation in phase 1 and 2 clinical trials testing the efficacy, safety, PK, and pharmacodynamics (PD) of AB928-based combination therapies for tumour indications (NCT03846310, NCT04262856, NCT04381832, NCT03720678, NCT04660812). AB928 can be orally administered by daily dosing. Promising preliminary results and results from previous studies suggest beneficial safety, PK, and PD profiles of the drug in patients [27, 29, 30].

Overall, AB928 reliably protected murine and human CAR T cells from all suppressive adenosine-mediated effects observed in this study, and when administered similarly to regimens currently under investigation in clinical trials, AB928 improved CAR T cell activation in vivo. Thus, we reason that the combination therapy with AB928 has high translational potential and may be a promising approach to enhance CAR T cell efficacy.

However, given the fact that other limitations of CAR T cell therapy such as limited trafficking to the tumour remain, we believe that multimodal approaches targeting more than one bottleneck of CAR T cell therapy are necessary to enable treatment of a broader range of tumours. We recently found that combined tumour-directed trafficking and expression of a dominant-negative receptor (DNR) to shield CAR T cells from TGF-β in the immunosuppressive TME synergistically improves CAR T cell efficacy in solid tumours [45]. Similar approaches combining tumour-directed recruitment and protection from the immunosuppressive TME may also be applicable for the combination therapy of AB928 and CAR T cells in the future.

## Supplementary information

Supplementary legend revised

Supplementary figure 1 revised

Supplementary figure 2 revised

Supplementary figure 3 revised

Supplementary figure 4 revised

Agreement of coauthors to changes in the authorlist

## Data Availability

The data sets generated and/or analysed during the current study available from the corresponding author on reasonable request.
