# Supplementary material for: Impact of the selective A2AR and A2BR dual antagonist AB928/etrumadenant on CAR T cell function
Source: Br J Cancer. 2022 Oct 20;127(12):2175–85. doi: 10.1038/s41416-022-02013-z (PMC9726885; doi:10.1038/s41416-022-02013-z)
Supplement: Supplementary file 1 — Supplementary legend revised [file 41416_2022_2013_MOESM1_ESM.docx]

**Figure S1: CAR T cell activation in the presence of AB928**

(**a**) Intracellular staining of murine anti‑EpCAM CAR T cells activated with plate bound recombinant EpCAM for 18 h in the presence or absence of NECA (1 µM) and AB928 (1 µM). Numbers represent the percentage of positively gated cells. (**b**) 10^5^ murine anti‑EpCAM CAR T cells were activated with Panc02‑EpCAM tumor cells or plate bound recombinant EpCAM for 18 h in the presence or absence of NECA (1 µM) and AB928 (1 µM). Surface expression of CD25 and CD69 were determined by flow cytometry at the end of the experiment. (**a**) Representative experiment of n = 3 independent experiments. Data are shown as representative contour plot. (**b**) Data are shown as mean ± SEM of n = 3 independent experiments with each dot representing the mean value of an individual experiment. *P < 0.05, **P < 0.01, ***P < 0.001 by one‑way ANOVA.

**Figure S2: CAR T cell effector responses and phenotype in the presence of AB928**

(**a**) RTCA of coculture with 5 x 10^4^ murine anti‑EpCAM CAR T cells and 2.5 x 10^4^ 4T1 tumor cells in the presence or absence of adenosine (10 µM + 2.5 µM EHNA) and AB928 (1 µM). CAR T cells and treatments were added at the timepoint indicated by the arrow. (**b**) Expression of activation markers on CAR T cells by flow cytometry. 10^5^ murine anti‑EpCAM CAR T cells were activated with Panc02‑EpCAM tumor cells or plate bound recombinant EpCAM for 18 h in the presence or absence of NECA (1 µM) and AB928 (1 µM). (**a**) Representative experiment of n = 3 independent experiments. Data represents mean of technical replicates. (**b**) Representative experiment of n = 3 independent experiments. Data are shown as representative contour plot.

**Figure S3: Schematic of human anti-mesothelin CAR constructs**

(**a**) Schematic representation of the human anti-mesothelin CAR constructs that differ in the transmembrane and intracellular domains.

**Figure S4: Human CAR T cell cytotoxicity and phenotype in the presence of NECA and AB928**

(**a**) RTCA of coculture with 2.5 x 10^4^ human anti‑MSLN-28z CAR T cells and 2.5 x 10^4^ SUIT-2-MSLN tumor cells in the presence or absence of NECA (1 µM) and AB928 (1 µM). CAR T cells and treatments were added at the timepoint indicated by the arrow. (**b**) Coculture 10^5^ anti‑MSLN‑CD28z CAR T cells and 2.5 x 10^4^ SUIT‑2‑MSLN tumor cells cells in the presence or absence of NECA (1 µM) and AB928 (1 µM). After 48 h phenotype of CD8^+^ CAR^+^ T cells was determined by flow cytometry. (**a**) Representative experiment of n = 3 independent experiments. Data represents mean of technical replicates. (**b**) Representative experiment of n = 4 independent experiments. Data are shown as representative contour plot.
