# Supplementary figures and images for: Impact of the selective A2AR and A2BR dual antagonist AB928/etrumadenant on CAR T cell function
Source: Br J Cancer. 2022 Oct 20;127(12):2175–85. doi: 10.1038/s41416-022-02013-z (PMC9726885; doi:10.1038/s41416-022-02013-z)

Supplementary figure 1

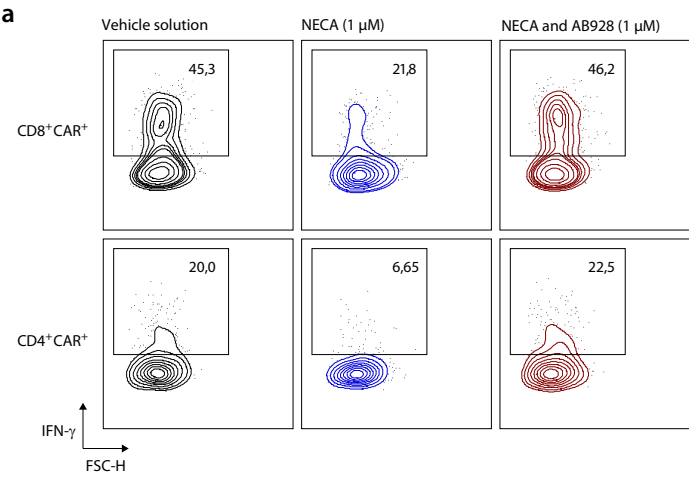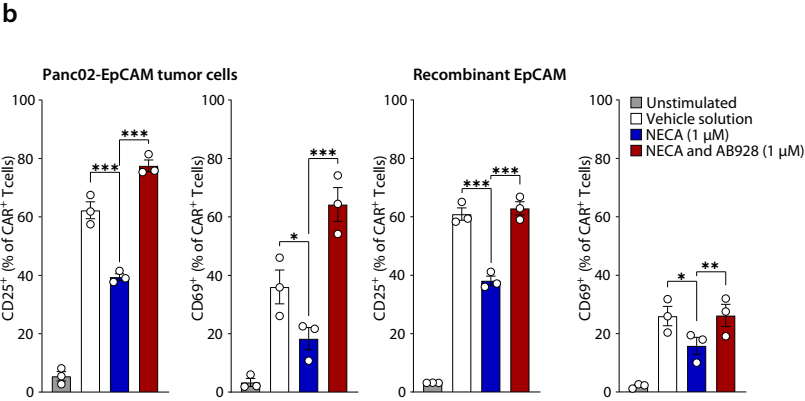

Supplement: Supplementary file 2 — Supplementary figure 1 revised [file 41416_2022_2013_MOESM2_ESM.pdf]

Supplementary figure 2

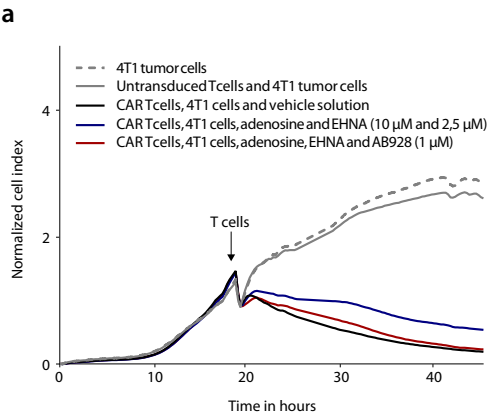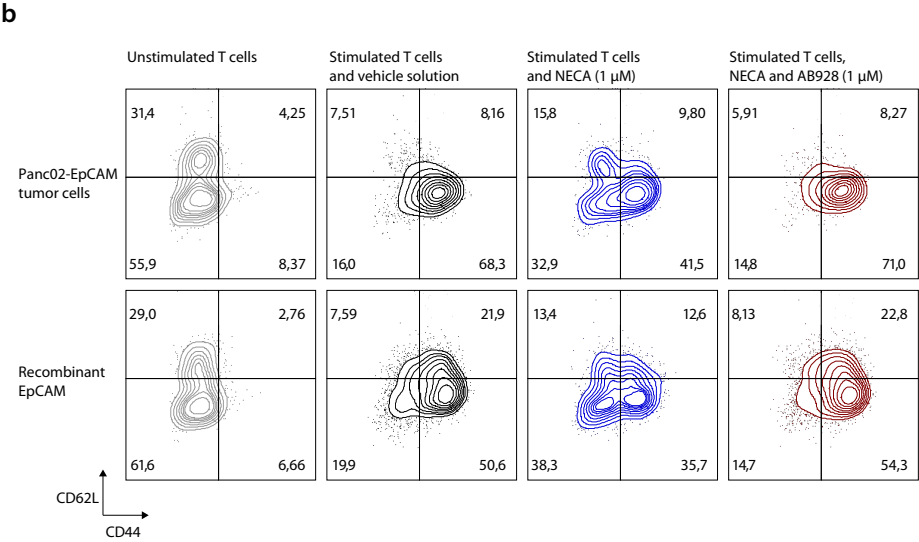

Supplement: Supplementary file 3 — Supplementary figure 2 revised [file 41416_2022_2013_MOESM3_ESM.pdf]

Supplementary figure 3

a

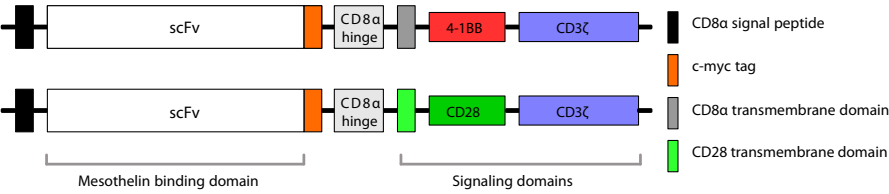

Supplement: Supplementary file 4 — Supplementary figure 3 revised [file 41416_2022_2013_MOESM4_ESM.pdf]

Supplementary Figure 4

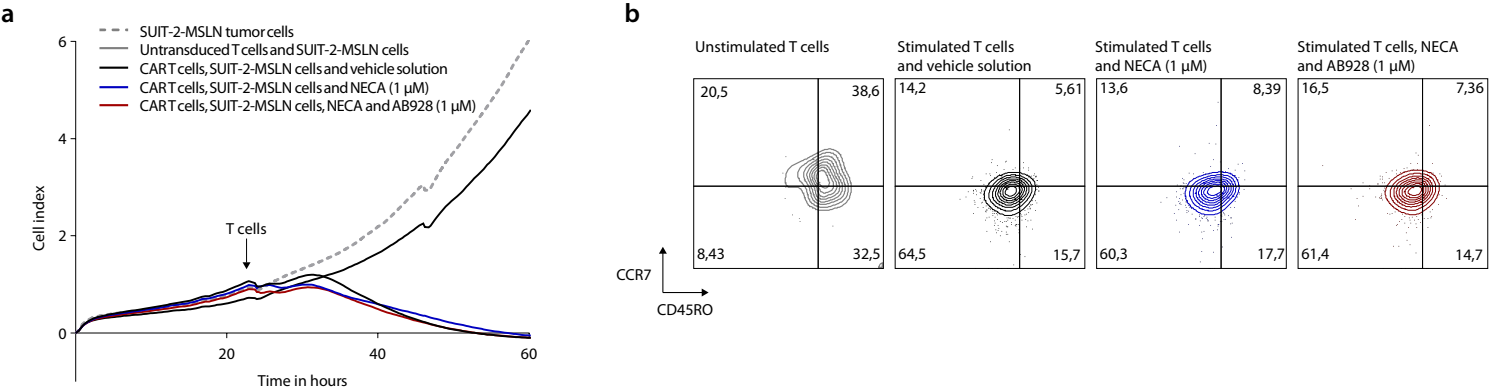

Supplement: Supplementary file 5 — Supplementary figure 4 revised [file 41416_2022_2013_MOESM5_ESM.pdf]
