# Supplementary material for: Impact of the selective A2AR and A2BR dual antagonist AB928/etrumadenant on CAR T cell function
Source: Br J Cancer. 2022 Oct 20;127(12):2175–85. doi: 10.1038/s41416-022-02013-z (PMC9726885; doi:10.1038/s41416-022-02013-z)
Supplement: Supplementary file 6 — Agreement of coauthors to changes in the authorlist [file 41416_2022_2013_MOESM6_ESM.pdf]

Gmail

Matthias Seifert <matti.seifert@gmail.com>

Change in authorlist of manuscript in BJC

19 Nachrichten

Matthias Seifert <matti.seifert@gmail.com> 22. September 2022 um 18:40
An: Sebastian Kobold <sebastian.kobold@med.uni-muenchen.de>, "Endres, Stefan Prof. Dr.med." <Stefan.Endres@med.uni-muenchen.de>, schindler.ulrike@t-online.de, Adrian Gottschlich <adrian.gottschlich@me.com>, 张瑾 <zhangjinnju@gmail.com>, Manouk Feinendegen <manouk@feinendegen.info>, Dario Dhoqina <dario.dhoqina@campus.lmu.de>, l.majed@campus.lmu.de, Hannah Obeck <hannah.obeck@gmail.com>, Öner Arman <armanoner@gmail.com>, Ruth Grünmeier <r.gruenmeier@gmx.de>, Theo Lorenzini <theo.lorenzini@campus.lmu.de>, David Andreu Sanz <David.Andreu@med.uni-muenchen.de>, "Stock, Sophia Carolina Denise Dr.med." <Sophia.Stock@med.uni-muenchen.de>, Jakob Jobst <jjj.jobst@t-online.de>, Bruno Cadilha <bcadilha@gmail.com>, "Dörr, Janina" <Janina.Doerr@med.uni-muenchen.de>, "Märkl, Florian" <Florian.Maerkl@med.uni-muenchen.de>, Daria Briukhovetska <daria.briukhovetska@gmail.com>, Reda Benmebarek <reda.benmebarek@gmail.com>

Dear co-authors,

The following authors have been added to the list of authors since the initial submission of our manuscript: Daria Briukhovetska, Florian Märkl, Sophia Stock, David Andreu-Sanz and Lina Majed.

The journal requests all co-authors agree to these changes. Please answer and confirm you agree as soon as possible (Also the authors added). A very brief answer will do.
(If you don't answer by September 29th, I assume you are in agreement with the changes, but please answer)

For reference find the email by the journal below:

3. It has come to our attention that your most recent author list differs from the one in your original submission. We find that the following authors have been added since your initial submission: Daria Briukhovetska, Florian Märkl, Sophia Stock, David Andreu-Sanz and Lina Majed. . Please request agreement from all authors, including the author being added in the following way:

Email your co-authors with the change, and ask them to reply to your email confirming that they agree to these changes. Once you have collected these replies, please combine all of the co-authors' email responses in one document and upload this file to your submission.

Thanks and best
Matthias

Theo Lorenzini <theo.lorenzini@campus.lmu.de> 22. September 2022 um 18:44
An: Matthias Seifert <matti.seifert@gmail.com>

Dear Matthias,

I agree to the change in the author list.

Best
Theo
[Zitierter Text ausgeblendet]

R.Gruenmeier@gmx.de <R.Gruenmeier@gmx.de> 22. September 2022 um 18:53
An: Matthias Seifert <matti.seifert@gmail.com>

I agree!
Viel Erfolg
Ruth

On 22.09.22 at 18:41, Matthias Seifert wrote:

From: "Matthias Seifert" <matti.seifert@gmail.com>
Date: 22. September 2022
To: "Bruno Cadilha" <bcadilha@gmail.com>,
"Daria Briukhovetska" <daria.briukhovetska@gmail.com>,
schindler.ulrike@t-online.de,
"张瑾" <zhangjinnju@gmail.com>,
"Adrian Gottschlich" <adrian.gottschlich@me.com>,
"Hannah Obeck" <hannah.obeck@gmail.com>,
"Öner Arman" <armanoner@gmail.com>,
"Märkl, Florian" <Florian.Maerkl@med.uni-muenchen.de>,
"Endres, Stefan Prof. Dr.med." <Stefan.Endres@med.uni-muenchen.de>,
"Jakob Jobst" <jjj.jobst@t-online.de>,
l.majed@campus.lmu.de,
"Dario Dhoqina" <dario.dhoqina@campus.lmu.de>,
"Sebastian Kobold" <sebastian.kobold@med.uni-muenchen.de>,
"Theo Lorenzini" <theo.lorenzini@campus.lmu.de>,
"David Andreu Sanz" <David.Andreu@med.uni-muenchen.de>,
"Ruth Grünmeier" <r.gruenmeier@gmx.de>,
"Dörr, Janina" <Janina.Doerr@med.uni-muenchen.de>,
"Reda Benmebarek" <reda.benmebarek@gmail.com>,
"Stock, Sophia Carolina Denise Dr.med." <Sophia.Stock@med.uni-muenchen.de>,
"Manouk Feinendegen" <manouk@feinendegen.info>
Cc:
Subject: Change in authorlist of manuscript in BJC
[Zitierter Text ausgeblendet]

Reda Benmebarek <reda.benmebarek@gmail.com> 22. September 2022 um 19:01
An: Matthias Seifert <matti.seifert@gmail.com>
Cc: Adrian Gottschlich <adrian.gottschlich@me.com>, Bruno Cadilha <bcadilha@gmail.com>, Daria Briukhovetska <daria.briukhovetska@gmail.com>, Dario Dhoqina <dario.dhoqina@campus.lmu.de>, David Andreu Sanz <David.Andreu@med.uni-muenchen.de>, "Dörr, Janina" <Janina.Doerr@med.uni-muenchen.de>, "Endres, Stefan Prof. Dr.med." <Stefan.Endres@med.uni-muenchen.de>, Hannah Obeck <hannah.obeck@gmail.com>, Jakob Jobst <jjj.jobst@t-online.de>, Manouk Feinendegen <manouk@feinendegen.info>, "Märkl, Florian" <Florian.Maerkl@med.uni-muenchen.de>, Ruth Grünmeier <r.gruenmeier@gmx.de>, Sebastian Kobold <sebastian.kobold@med.uni-muenchen.de>, "Stock, Sophia Carolina Denise Dr.med." <Sophia.Stock@med.uni-muenchen.de>, Theo Lorenzini <theo.lorenzini@campus.lmu.de>, l.majed@campus.lmu.de, schindler.ulrike@t-online.de, Öner Arman <armanoner@gmail.com>, 张瑾 <zhangjinnju@gmail.com>

Yes, I agree.
[Zitierter Text ausgeblendet]

Endres, Stefan Prof. Dr.med. <Stefan.Endres@med.uni-muenchen.de> 22. September 2022 um 19:14
An: Matthias Seifert <matti.seifert@gmail.com>

My agreement

Stefan Endres

Prof. Dr. med. Stefan Endres

LMU Klinikum

Abteilung für Klinische Pharmakologie

[Zitierter Text ausgeblendet]

Bruno Cadilha <bcadilha@gmail.com> 22. September 2022 um 19:14
An: Reda Benmebarek <reda.benmebarek@gmail.com>
Cc: Adrian Gottschlich <adrian.gottschlich@me.com>, Daria Briukhovetska <daria.briukhovetska@gmail.com>, Dario Dhoqina <dario.dhoqina@campus.lmu.de>, David Andreu Sanz <David.Andreu@med.uni-muenchen.de>, "Dörr, Janina" <Janina.Doerr@med.uni-muenchen.de>, "Endres, Stefan Prof. Dr.med." <Stefan.Endres@med.uni-muenchen.de>, Hannah Obeck <hannah.obeck@gmail.com>, Jakob Jobst <jjj.jobst@t-online.de>, Manouk Feinendegen <manouk@feinendegen.info>, Matthias Seifert <matti.seifert@gmail.com>, "Märkl, Florian" <Florian.Maerkl@med.uni-muenchen.de>, Ruth Grünmeier <r.gruenmeier@gmx.de>, Sebastian Kobold <sebastian.kobold@med.uni-muenchen.de>, "Stock, Sophia Carolina Denise Dr.med." <Sophia.Stock@med.uni-muenchen.de>, Theo Lorenzini <theo.lorenzini@campus.lmu.de>, l.majed@campus.lmu.de, schindler.ulrike@t-online.de, Öner Arman <armanoner@gmail.com>, 张瑾 <zhangjinnju@gmail.com>

I agree.
Kind regards
Bruno
[Zitierter Text ausgeblendet]

Arman Öner <armanoner@gmail.com> 22. September 2022 um 19:35
An: Bruno Cadilha <bcadilha@gmail.com>
Cc: Adrian Gottschlich <adrian.gottschlich@me.com>, Daria Briukhovetska <daria.briukhovetska@gmail.com>, Dario Dhoqina <dario.dhoqina@campus.lmu.de>, David Andreu Sanz <David.Andreu@med.uni-muenchen.de>, "Dörr, Janina" <Janina.Doerr@med.uni-muenchen.de>, "Endres, Stefan Prof. Dr.med." <Stefan.Endres@med.uni-muenchen.de>, Hannah Obeck <hannah.obeck@gmail.com>, Jakob Jobst <jjj.jobst@t-online.de>, Manouk Feinendegen <manouk@feinendegen.info>, Matthias Seifert <matti.seifert@gmail.com>, "Märkl, Florian" <Florian.Maerkl@med.uni-muenchen.de>, Reda Benmebarek <reda.benmebarek@gmail.com>, Ruth Grünmeier <r.gruenmeier@gmx.de>, Sebastian Kobold <sebastian.kobold@med.uni-muenchen.de>, "Stock, Sophia Carolina Denise Dr.med." <Sophia.Stock@med.uni-muenchen.de>, Theo Lorenzini <theo.lorenzini@campus.lmu.de>, l.majed@campus.lmu.de, schindler.ulrike@t-online.de, 张瑾 <zhangjinnju@gmail.com>

I agree as well.

Regards

Arman
[Zitierter Text ausgeblendet]

Univ.- Prof. Dr. med. Sebastian Kobold <sebastian.kobold@med.uni-muenchen.de> 22. September 2022 um 19:40
An: Matthias Seifert <matti.seifert@gmail.com>

Hi jakob, i of course agree. Thanks seb

Univ.-Prof. Dr. med. Sebastian Kobold

Abteilung für Klinische Pharmakologie
Klinikum der Universität München
Lindwurmstrasse 2a
80337 München

- send from a mobile device, please excuse my brievity and any unintentional typos -

-------- Ursprüngliche Nachricht --------
Von: Matthias Seifert <matti.seifert@gmail.com>
Datum: 22.09.22 18:41 (GMT+01:00)
An: Sebastian Kobold <sebastian.kobold@med.uni-muenchen.de>, "Endres, Stefan Prof. Dr.med." <Stefan.Endres@med.uni-muenchen.de>, schindler.ulrike@t-online.de, Adrian Gottschlich <adrian.gottschlich@me.com>, 张瑾 <zhangjinnju@gmail.com>, Manouk Feinendegen

<manouk@feinendegen.info>, Dario Dhoqina <dario.dhoqina@campus.lmu.de>, l.majed@campus.lmu.de, Hannah Obeck <hannah.obeck@gmail.com>, Öner Arman <armanoner@gmail.com>, Ruth Grünmeier <r.gruenmeier@gmx.de>, Theo Lorenzini <theo.lorenzini@campus.lmu.de>, David Andreu Sanz <David.Andreu@med.uni-muenchen.de>, "Stock, Sophia Carolina Denise Dr.med." <Sophia.Stock@med.uni-muenchen.de>, Jakob Jobst <jjj.jobst@t-online.de>, Bruno Cadilha <bcadilha@gmail.com>, "Dörr, Janina" <Janina.Doerr@med.uni-muenchen.de>, "Märkl, Florian" <Florian.Maerkl@med.uni-muenchen.de>, Daria Briukhovetska <daria.briukhovetska@gmail.com>, Reda Benmebarek <reda.benmebarek@gmail.com>
Betreff: Change in authorlist of manuscript in BJC

[Zitierter Text ausgeblendet]

David Andreu Sanz <david.andreu@med.uni-muenchen.de> 22. September 2022 um 19:52
An: Matthias Seifert <matti.seifert@gmail.com>, Sebastian Kobold <sebastian.kobold@med.uni-muenchen.de>, "Endres, Stefan Prof. Dr.med." <Stefan.Endres@med.uni-muenchen.de>, schindler.ulrike@t-online.de, Adrian Gottschlich <adrian.gottschlich@me.com>, 张瑾 <zhangjinnju@gmail.com>, Manouk Feinendegen <manouk@feinendegen.info>, Dario Dhoqina <dario.dhoqina@campus.lmu.de>, l.majed@campus.lmu.de, Hannah Obeck <hannah.obeck@gmail.com>, Öner Arman <armanoner@gmail.com>, Ruth Grünmeier <r.gruenmeier@gmx.de>, Theo Lorenzini <theo.lorenzini@campus.lmu.de>, "Stock, Sophia Carolina Denise Dr.med." <Sophia.Stock@med.uni-muenchen.de>, Jakob Jobst <jjj.jobst@t-online.de>, Bruno Cadilha <bcadilha@gmail.com>, "Dörr, Janina" <Janina.Doerr@med.uni-muenchen.de>, "Märkl, Florian" <Florian.Maerkl@med.uni-muenchen.de>, Daria Briukhovetska <daria.briukhovetska@gmail.com>, Reda Benmebarek <reda.benmebarek@gmail.com>

Yes, I agree.

Best regards,

David

[Zitierter Text ausgeblendet]

---

proexe230298@gmail.com <schindler.ulrike@t-online.de> 22. September 2022 um 20:10
An: David Andreu Sanz <david.andreu@med.uni-muenchen.de>, Matthias Seifert <matti.seifert@gmail.com>, Sebastian Kobold <sebastian.kobold@med.uni-muenchen.de>, "Endres, Stefan Prof. Dr.med." <Stefan.Endres@med.uni-muenchen.de>, Adrian Gottschlich <adrian.gottschlich@me.com>, 张瑾 <zhangjinnju@gmail.com>, Manouk Feinendegen <manouk@feinendegen.info>, Dario Dhoqina <dario.dhoqina@campus.lmu.de>, l.majed@campus.lmu.de, Hannah Obeck <hannah.obeck@gmail.com>, Öner Arman <armanoner@gmail.com>, Ruth Grünmeier <r.gruenmeier@gmx.de>, Theo Lorenzini <theo.lorenzini@campus.lmu.de>, "Stock, Sophia Carolina Denise Dr.med." <Sophia.Stock@med.uni-muenchen.de>, Jakob Jobst <jjj.jobst@t-online.de>, Bruno Cadilha <bcadilha@gmail.com>, "Dörr, Janina" <Janina.Doerr@med.uni-muenchen.de>, "Märkl, Florian" <Florian.Maerkl@med.uni-muenchen.de>, Daria Briukhovetska <daria.briukhovetska@gmail.com>, Reda Benmebarek <reda.benmebarek@gmail.com>

Yes, I also agree.

Thanks, Uli

Gesendet mit der Telekom Mail App

-----Original-Nachricht-----
Von: David Andreu Sanz <david.andreu@med.uni-muenchen.de>
Betreff: RE: Change in authorlist of manuscript in BJC
Datum: 22.09.2022, 19:52 Uhr
An: 'Matthias Seifert' <matti.seifert@gmail.com>, 'Sebastian Kobold' <sebastian.kobold@med.uni-muenchen.de>, 'Endres, Stefan Prof. Dr.med.' <Stefan.Endres@med.uni-muenchen.de>, <schindler.ulrike@t-online.de>, 'Adrian Gottschlich' <adrian.gottschlich@me.com>, '张瑾' <zhangjinnju@gmail.com>, 'Manouk Feinendegen' <manouk@feinendegen.info>, 'Dario Dhoqina' <dario.dhoqina@campus.lmu.de>, <l.majed@campus.lmu.de>, 'Hannah Obeck' <hannah.obeck@gmail.com>, 'Öner Arman' <armanoner@gmail.com>, 'Ruth Grünmeier' <r.gruenmeier@gmx.de>, 'Theo Lorenzini' <theo.lorenzini@campus.lmu.de>, 'Stock, Sophia Carolina Denise Dr.med.' <Sophia.Stock@med.uni-muenchen.de>, 'Jakob Jobst' <jjj.jobst@t-online.de>, 'Bruno Cadilha' <bcadilha@gmail.com>, 'Dörr, Janina' <Janina.Doerr@med.uni-muenchen.de>, 'Märkl, Florian' <Florian.Maerkl@med.uni-muenchen.de>, 'Daria Briukhovetska' <daria.briukhovetska@gmail.com>, 'Reda Benmebarek' <reda.benmebarek@gmail.com>

[Zitierter Text ausgeblendet]

Dario Dhoqina <Dario.Dhoqina@campus.lmu.de> 22. September 2022 um 20:16
An: Matthias Seifert <matti.seifert@gmail.com>
Cc: Sebastian Kobold <sebastian.kobold@med.uni-muenchen.de>, "Endres, Stefan Prof. Dr.med." <Stefan.Endres@med.uni-muenchen.de>, schindler.ulrike@t-online.de, Adrian Gottschlich <adrian.gottschlich@me.com>, 张瑾 <zhangjinnju@gmail.com>, Manouk Feinendegen <manouk@feinendegen.info>, l.majed@campus.lmu.de, Hannah Obeck <hannah.obeck@gmail.com>, Öner Arman <armanoner@gmail.com>, Ruth Grünmeier <r.gruenmeier@gmx.de>, Theo Lorenzini <theo.lorenzini@campus.lmu.de>, David Andreu Sanz <David.Andreu@med.uni-muenchen.de>, "Stock, Sophia Carolina Denise Dr.med." <Sophia.Stock@med.uni-muenchen.de>, Jakob Jobst <jjj.jobst@t-online.de>, Bruno Cadilha <bcadilha@gmail.com>, "\"Dörr, Janina\"" <Janina.Doerr@med.uni-muenchen.de>, "\"Märkl, Florian\"" <Florian.Maerkl@med.uni-muenchen.de>, Daria Briukhovetska <daria.briukhovetska@gmail.com>, Reda Benmebarek <reda.benmebarek@gmail.com>

Yes, I agree to the changes.

Best regards,
Dario

[Zitierter Text ausgeblendet]

Stock, Sophia Carolina Denise Dr.med. <Sophia.Stock@med.uni-muenchen.de> 22. September 2022 um 21:05
An: Matthias Seifert <matti.seifert@gmail.com>, "Kobold, Sebastian Prof. Dr." <Sebastian.Kobold@med.uni-muenchen.de>, "Endres, Stefan Prof. Dr.med." <Stefan.Endres@med.uni-muenchen.de>, "schindler.ulrike@t-online.de" <schindler.ulrike@t-online.de>, Adrian Gottschlich <adrian.gottschlich@me.com>, 张瑾 <zhangjinnju@gmail.com>, Manouk Feinendegen <manouk@feinendegen.info>, Dario Dhoqina <dario.dhoqina@campus.lmu.de>, "l.majed@campus.lmu.de" <l.majed@campus.lmu.de>, Hannah Obeck <hannah.obeck@gmail.com>, Öner Arman <armanoner@gmail.com>, Ruth Grünmeier <r.gruenmeier@gmx.de>, Theo Lorenzini <theo.lorenzini@campus.lmu.de>, "Andreu Sanz, David" <David.Andreu@med.uni-muenchen.de>, Jakob Jobst <jjj.jobst@t-online.de>, Bruno Cadilha <bcadilha@gmail.com>, "Dörr, Janina" <Janina.Doerr@med.uni-muenchen.de>, "Märkl, Florian" <Florian.Maerkl@med.uni-muenchen.de>, Daria Briukhovetska <daria.briukhovetska@gmail.com>, Reda Benmebarek <reda.benmebarek@gmail.com>

I agree.

Best,

Sophia

Von: Matthias Seifert <matti.seifert@gmail.com>
Gesendet: Donnerstag, 22. September 2022 18:40
An: Kobold, Sebastian Prof. Dr.; Endres, Stefan Prof. Dr.med.; schindler.ulrike@t-online.de; Adrian Gottschlich; 张瑾; Manouk Feinendegen; Dario Dhoqina; l.majed@campus.lmu.de; Hannah Obeck; Öner Arman; Ruth Grünmeier; Theo Lorenzini; Andreu Sanz, David; Stock, Sophia Carolina Denise Dr.med.; Jakob Jobst; Bruno Cadilha; Dörr, Janina; Märkl, Florian; Daria Briukhovetska; Reda Benmebarek
Betreff: Change in authorlist of manuscript in BJC

[Zitierter Text ausgeblendet]

Hannah Obeck <hannah.obeck@gmail.com> 22. September 2022 um 22:19
An: Arman Öner <armanoner@gmail.com>
Cc: Bruno Cadilha <bcadilha@gmail.com>, Adrian Gottschlich <adrian.gottschlich@me.com>, Daria Briukhovetska <daria.briukhovetska@gmail.com>, Dario Dhoqina <dario.dhoqina@campus.lmu.de>, David Andreu Sanz <David.Andreu@med.uni-muenchen.de>, "\"Dörr, Janina\"" <Janina.Doerr@med.uni-muenchen.de>, "Endres, Stefan Prof. Dr.med." <Stefan.Endres@med.uni-muenchen.de>, Jakob Jobst <jjj.jobst@t-online.de>, Manouk Feinendegen <manouk@feinendegen.info>, Matthias Seifert <matti.seifert@gmail.com>, "\"Märkl, Florian\"" <Florian.Maerkl@med.uni-muenchen.de>, Reda Benmebarek <reda.benmebarek@gmail.com>, Ruth Grünmeier <r.gruenmeier@gmx.de>, Sebastian Kobold <sebastian.kobold@med.uni-muenchen.de>, "Stock, Sophia Carolina Denise Dr.med." <Sophia.Stock@med.uni-muenchen.de>, Theo Lorenzini <theo.lorenzini@campus.lmu.de>, l.majed@campus.lmu.de, schindler.ulrike@t-online.de, 张瑾 <zhangjinnju@gmail.com>

I agree too.

Best

Hannah

Von meinem iPhone gesendet

Am 22.09.2022 um 19:35 schrieb Arman Öner <armanoner@gmail.com>:

[Zitierter Text ausgeblendet]

Daria Briukhovetska <daria.briukhovetska@gmail.com> 22. September 2022 um 22:20
An: "Stock, Sophia Carolina Denise Dr.med." <Sophia.Stock@med.uni-muenchen.de>
Cc: Matthias Seifert <matti.seifert@gmail.com>, "Kobold, Sebastian Prof. Dr." <Sebastian.Kobold@med.uni-muenchen.de>, "Endres, Stefan Prof. Dr.med." <Stefan.Endres@med.uni-muenchen.de>, schindler.ulrike@t-online.de, Adrian Gottschlich <adrian.gottschlich@me.com>, 张瑾 <zhangjinnju@gmail.com>, Manouk Feinendegen <manouk@feinendegen.info>, Dario Dhoqina <dario.dhoqina@campus.lmu.de>, l.majed@campus.lmu.de, Hannah Obeck <hannah.obeck@gmail.com>, Öner Arman <armanoner@gmail.com>, Ruth Grünmeier <r.gruenmeier@gmx.de>, Theo Lorenzini <theo.lorenzini@campus.lmu.de>, "Andreu Sanz, David" <David.Andreu@med.uni-muenchen.de>, Jakob Jobst <jjj.jobst@t-online.de>, Bruno Cadilha <bcadilha@gmail.com>, "Dörr, Janina" <Janina.Doerr@med.uni-muenchen.de>, "Märkl, Florian" <Florian.Maerkl@med.uni-muenchen.de>, Reda Benmebarek <reda.benmebarek@gmail.com>

Me too
Best
Daria
[Zitierter Text ausgeblendet]

Lina Majed <L.Majed@campus.lmu.de> 23. September 2022 um 00:11
An: Matthias Seifert <matti.seifert@gmail.com>
Cc: Sebastian Kobold <sebastian.kobold@med.uni-muenchen.de>, "Endres, Stefan Prof. Dr.med." <Stefan.Endres@med.uni-muenchen.de>, schindler.ulrike@t-online.de, Adrian Gottschlich <adrian.gottschlich@me.com>, 张瑾 <zhangjinnju@gmail.com>, Manouk Feinendegen <manouk@feinendegen.info>, Dario Dhoqina <dario.dhoqina@campus.lmu.de>, l.majed@campus.lmu.de, Hannah Obeck <hannah.obeck@gmail.com>, Öner Arman <armanoner@gmail.com>, Ruth Grünmeier <r.gruenmeier@gmx.de>, Theo Lorenzini <theo.lorenzini@campus.lmu.de>, David Andreu Sanz <David.Andreu@med.uni-muenchen.de>, "Stock, Sophia Carolina Denise Dr.med." <Sophia.Stock@med.uni-muenchen.de>, Jakob Jobst <jjj.jobst@t-online.de>, Bruno Cadilha <bcadilha@gmail.com>, "Dörr, Janina" <Janina.Doerr@med.uni-muenchen.de>, "Märkl, Florian" <Florian.Maerkl@med.uni-muenchen.de>, Daria Briukhovetska <daria.briukhovetska@gmail.com>, Reda Benmebarek <reda.benmebarek@gmail.com>

I agree as well.

Best regards,

Lina

> Ursprüngliche Nachricht:
> Von: Matthias Seifert <matti.seifert@gmail.com>
> An: Sebastian Kobold <sebastian.kobold@med.uni-muenchen.de>, "Endres, Stefan Prof.
> Dr.med." <Stefan.Endres@med.uni-muenchen.de>, schindler.ulrike@t-online.de, Adrian
> Gottschlich <adrian.gottschlich@me.com>, "张瑾" <zhangjinnju@gmail.com>, Manouk Feinendegen
> <manouk@feinendegen.info>, Dario Dhoqina <dario.dhoqina@campus.lmu.de>, l.majed@campus.lmu.de,
> Hannah Obeck <hannah.obeck@gmail.com>, "Öner Arman" <armanoner@gmail.com>, "Ruth
> Grünmeier" <r.gruenmeier@gmx.de>, Theo Lorenzini <theo.lorenzini@campus.lmu.de>,
> David Andreu Sanz <David.Andreu@med.uni-muenchen.de>, "Stock, Sophia Carolina Denise
> Dr.med." <Sophia.Stock@med.uni-muenchen.de>, Jakob Jobst <jjj.jobst@t-online.de>,
> Bruno Cadilha <bcadilha@gmail.com>, "Dörr, Janina" <Janina.Doerr@med.uni-muenchen.de>,
> "Märkl, Florian" <Florian.Maerkl@med.uni-muenchen.de>, Daria Briukhovetska <daria.briukhovetska
> @gmail.com>, Reda Benmebarek <reda.benmebarek@gmail.com>
> Kopie:
> Datum: Thu Sep 22 18:41:02 CEST 2022
>

> Dear co-authors,
>
> The following authors have been added to the list of authors since the
> initial submission of our manuscript: Daria Briukhovetska, Florian Märkl,
> Sophia Stock, David Andreu-Sanz and Lina Majed.
>
> The journal requests all co-authors agree to these changes. Please answer
> and confirm you agree as soon as possible (Also the authors added). A very
> brief answer will do.
> (If you don't answer by September 29th, I assume you are in agreement with
> the changes, but please answer)
>
> For reference find the email by the journal below:
>
>
>
> *3. It has come to our attention that your most recent author list differs
> from the one in your original submission. We find that the following
> authors have been added since your initial submission: Daria Briukhovetska,
> Florian Märkl, Sophia Stock, David Andreu-Sanz and Lina Majed. . Please
> request agreement from all authors, including the author being added in the
> following way:Email your co-authors with the change, and ask them to reply
> to your email confirming that they agree to these changes. Once you have
> collected these replies, please combine all of the co-authors' email
> responses in one document and upload this file to your submission.*
>
> Thanks and best
> Matthias
>

Jakob Jobst <jjj.jobst@t-online.de> 23. September 2022 um 01:34
An: schindler.ulrike@t-online.de
Cc: David Andreu Sanz <David.Andreu@med.uni-muenchen.de>, Matthias Seifert <matti.seifert@gmail.com>, Sebastian Kobold <sebastian.kobold@med.uni-muenchen.de>, "Endres, Stefan Prof. Dr.med." <Stefan.Endres@med.uni-muenchen.de>, Adrian Gottschlich <adrian.gottschlich@me.com>, 张瑾 <zhangjinnju@gmail.com>, Manouk Feinendegen <manouk@feinendegen.info>, Dario Dhoqina <dario.dhoqina@campus.lmu.de>, l.majed@campus.lmu.de, Hannah Obeck <hannah.obeck@gmail.com>, Öner Arman <armanoner@gmail.com>, Ruth Grünmeier <r.gruenmeier@gmx.de>, Theo Lorenzini <theo.lorenzini@campus.lmu.de>, "Stock, Sophia Carolina Denise Dr.med." <Sophia.Stock@med.uni-muenchen.de>, Bruno Cadilha <bcadilha@gmail.com>, "\"Dörr, Janina\"" <Janina.Doerr@med.uni-muenchen.de>, "\"Märkl, Florian\"" <Florian.Maerkl@med.uni-muenchen.de>, Daria Briukhovetska <daria.briukhovetska@gmail.com>, Reda Benmebarek <reda.benmebarek@gmail.com>

I agree as well.

Best
Jakob

Am 22.09.2022 um 20:10 schrieb schindler.ulrike@t-online.de:

Yes, I also agree.

Thanks, Uli

Gesendet mit der Telekom Mail App

-----Original-Nachricht-----
Von: David Andreu Sanz <david.andreu@med.uni-muenchen.de>
Betreff: RE: Change in authorlist of manuscript in BJC
Datum: 22.09.2022, 19:52 Uhr
An: 'Matthias Seifert' <matti.seifert@gmail.com>, 'Sebastian Kobold' <sebastian.kobold@med.uni-muenchen.de>, 'Endres, Stefan Prof. Dr.med.' <Stefan.Endres@med.uni-muenchen.de>, <schindler.ulrike@t-online.de>, 'Adrian Gottschlich' <adrian.gottschlich@me.com>, '张瑾'

<zhangjinnju@gmail.com>, 'Manouk Feinendegen' <manouk@feinendegen.info>, 'Dario Dhoqina' <dario.dhoqina@campus.lmu.de>, <l.majed@campus.lmu.de>, 'Hannah Obeck' <hannah.obeck@gmail.com>, 'Öner Arman' <armanoner@gmail.com>, 'Ruth Grünmeier' <r.gruenmeier@gmx.de>, 'Theo Lorenzini' <theo.lorenzini@campus.lmu.de>, 'Stock, Sophia Carolina Denise Dr.med.' <Sophia.Stock@med.uni-muenchen.de>, 'Jakob Jobst' <jjj.jobst@t-online.de>, 'Bruno Cadilha' <bcadilha@gmail.com>, 'Dörr, Janina' <Janina.Doerr@med.uni-muenchen.de>, 'Märkl, Florian' <Florian.Maerkl@med.uni-muenchen.de>, 'Daria Briukhovetska' <daria.briukhovetska@gmail.com>, 'Reda Benmebarek' <reda.benmebarek@gmail.com>

Yes, I agree.

Best regards,

David

From: Matthias Seifert <matti.seifert@gmail.com>
Sent: jueves, 22 de septiembre de 2022 18:41
To: Sebastian Kobold <sebastian.kobold@med.uni-muenchen.de>; Endres, Stefan Prof. Dr.med. <Stefan.Endres@med.uni-muenchen.de>; schindler.ulrike@t-online.de; Adrian Gottschlich <adrian.gottschlich@me.com>; 张瑾 <zhangjinnju@gmail.com>; Manouk Feinendegen <manouk@feinendegen.info>; Dario Dhoqina <dario.dhoqina@campus.lmu.de>; l.majed@campus.lmu.de; Hannah Obeck <hannah.obeck@gmail.com>; Öner Arman <armanoner@gmail.com>; Ruth Grünmeier <r.gruenmeier@gmx.de>; Theo Lorenzini <theo.lorenzini@campus.lmu.de>; David Andreu Sanz <David.Andreu@med.uni-muenchen.de>; Stock, Sophia Carolina Denise Dr.med. <Sophia.Stock@med.uni-muenchen.de>; Jakob Jobst <jjj.jobst@t-online.de>; Bruno Cadilha <bcadilha@gmail.com>; Dörr, Janina <Janina.Doerr@med.uni-muenchen.de>; Märkl, Florian <Florian.Maerkl@med.uni-muenchen.de>; Daria Briukhovetska <daria.briukhovetska@gmail.com>; Reda Benmebarek <reda.benmebarek@gmail.com>
Subject: Change in authorlist of manuscript in BJC

Dear co-authors,

The following authors have been added to the list of authors since the initial submission of our manuscript: Daria Briukhovetska, Florian Märkl, Sophia Stock, David Andreu-Sanz and Lina Majed.

The journal requests all co-authors agree to these changes. Please answer and confirm you agree as soon as possible (Also the authors added). A very brief answer will do.

(If you don't answer by September 29th, I assume you are in agreement with the changes, but please answer)

For reference find the email by the journal below:

3. It has come to our attention that your most recent author list differs from the one in your original submission. We find that the following authors have been added since your initial submission: Daria Briukhovetska, Florian Märkl, Sophia Stock, David Andreu-Sanz and Lina Majed. . Please request agreement from all authors, including the author being added in the following way:

Email your co-authors with the change, and ask them to reply to your email confirming that they agree to these changes. Once you have collected these replies, please combine all of the co-authors' email responses in one document and upload this file to your submission.

Thanks and best

Matthias

Märkl, Florian <Florian.Maerkl@med.uni-muenchen.de> 23. September 2022 um 09:20
An: Matthias Seifert <matti.seifert@gmail.com>, "Kobold, Sebastian Prof. Dr." <Sebastian.Kobold@med.uni-muenchen.de>, "Endres, Stefan Prof. Dr.med." <Stefan.Endres@med.uni-muenchen.de>, "schindler.ulrike@t-online.de" <schindler.ulrike@t-online.de>, Adrian Gottschlich <adrian.gottschlich@me.com>, 张瑾 <zhangjinnju@gmail.com>, Manouk Feinendegen <manouk@feinendegen.info>, Dario Dhoqina <dario.dhoqina@campus.lmu.de>, "l.majed@campus.lmu.de" <l.majed@campus.lmu.de>, Hannah Obeck <hannah.obeck@gmail.com>, Öner Arman <armanoner@gmail.com>, Ruth Grünmeier <r.gruenmeier@gmx.de>, Theo Lorenzini <theo.lorenzini@campus.lmu.de>, "Andreu Sanz, David" <David.Andreu@med.uni-muenchen.de>, "Stock, Sophia Carolina Denise Dr.med." <Sophia.Stock@med.uni-muenchen.de>, Jakob Jobst <jjj.jobst@t-online.de>, Bruno Cadilha <bcadilha@gmail.com>, "Dörr, Janina" <Janina.Doerr@med.uni-muenchen.de>, Daria Briukhovetska <daria.briukhovetska@gmail.com>, Reda Benmebarek <reda.benmebarek@gmail.com>

I agree.

Best,

Florian

Dörr, Janina <Janina.Doerr@med.uni-muenchen.de> 26. September 2022 um 10:19
An: Matthias Seifert <matti.seifert@gmail.com>

Hi,
I agree!
Best, Janina

Janina Dörr
AG Kobold
Abteilung für Klinische Pharmakologie
Klinikum der Universität München
Lindwurmstr. 2A, 80337 München

Von: Matthias Seifert <matti.seifert@gmail.com>
Gesendet: Donnerstag, 22. September 2022 18:40:50
An: Kobold, Sebastian Prof. Dr.; Endres, Stefan Prof. Dr.med.; schindler.ulrike@t-online.de; Adrian Gottschlich; 张瑾; Manouk Feinendegen; Dario Dhoqina; l.majed@campus.lmu.de; Hannah Obeck; Öner Arman; Ruth Grünmeier; Theo Lorenzini; Andreu Sanz, David; Stock, Sophia Carolina Denise Dr.med.; Jakob Jobst; Bruno Cadilha; Dörr, Janina; Märkl, Florian; Daria Briukhovetska; Reda Benmebarek
Betreff: Change in authorlist of manuscript in BJC

[Zitierter Text ausgeblendet]

Manouk Feinendegen <manouk@feinendegen.info> 26. September 2022 um 11:52
An: Matthias Seifert <matti.seifert@gmail.com>

Yes, I agree.
Thanks
Manouk

> Matthias Seifert <matti.seifert@gmail.com> hat am 22.09.2022 18:40 CEST geschrieben:
>
>
> Dear co-authors,
>
> The following authors have been added to the list of authors since the

> initial submission of our manuscript: Daria Briukhovetska, Florian Märkl,
> Sophia Stock, David Andreu-Sanz and Lina Majed.
>
> The journal requests all co-authors agree to these changes. Please answer
> and confirm you agree as soon as possible (Also the authors added). A very
> brief answer will do.
> (If you don't answer by September 29th, I assume you are in agreement with
> the changes, but please answer)
>
> For reference find the email by the journal below:
>
>
>
> *3. It has come to our attention that your most recent author list differs
> from the one in your original submission. We find that the following
> authors have been added since your initial submission: Daria Briukhovetska,
> Florian Märkl, Sophia Stock, David Andreu-Sanz and Lina Majed. . Please
> request agreement from all authors, including the author being added in the
> following way:Email your co-authors with the change, and ask them to reply
> to your email confirming that they agree to these changes. Once you have
> collected these replies, please combine all of the co-authors' email

[Zitierter Text ausgeblendet]

Note: Jin Zhang was not reachable despite multiple attemps. We assume she agrees with the changes to the authorlist
